# Regulating Morphology and Composition of Laser-Induced Periodic Structures on Titanium Films with Femtosecond Laser Wavelength and Ambient Environment

**DOI:** 10.3390/nano12030306

**Published:** 2022-01-18

**Authors:** Kirill Bronnikov, Semyon Gladkikh, Konstantin Okotrub, Andrey Simanchuk, Alexey Zhizhchenko, Aleksandr Kuchmizhak, Alexander Dostovalov

**Affiliations:** 1 Institute of Automation and Electrometry of the SB RAS, 1 Acad. Koptyug Ave., 630090 Novosibirsk, Russia; s.gladkikh@g.nsu.ru (S.G.); okotrubk@gmail.com (K.O.); simmk@yandex.ru (A.S.); alexdost@gmail.com (A.D.); 2 Institute of Automation and Control Processes of the FEB RAS, 5 Radio St., 690041 Vladivostok, Russia; g89leksig@mail.ru (A.Z.); alex.iacp.dvo@mail.ru (A.K.); 3 Far Eastern Federal University, 690041 Vladivostok, Russia; 4 Pacific Quantum Center, Far Eastern Federal University, 690041 Vladivostok, Russia

**Keywords:** laser-induced periodic surface structures, femtosecond laser pulses, thin films, titanium nitride

## Abstract

Recently, highly uniform thermochemical laser-induced periodic surface structures (TLIPSS) have attracted significant research attention due to their practical applicability for upscalable fabrication of periodic surface morphologies important for surface functionalization, diffraction optics, sensors, etc. When processed by femtosecond (fs) laser pulses in oxygen-containing environments, TLIPSS are formed on the material surface as parallel protrusions upon local oxidation in the maxima of the periodic intensity pattern coming from interference of the incident and scattered waves. From an application point of view, it is important to control both the TLIPSS period and nanoscale morphology of the formed protrusions that can be expectedly achieved by scalable shrinkage of the laser-processing wavelength as well as by varying the ambient environment. However, so far, the fabrication of uniform TLIPSS was reported only for near-IR wavelength in air. In this work, TLIPSS formation on the surface of titanium (Ti) films was systematically studied using near-IR (1026 nm), visible (513 nm) and UV (256 nm) wavelengths revealing linear scalability of the protrusion period versus the fs-laser wavelength. By changing the ambient environment from air to vacuum (10^−2^ atm) and pressurized nitrogen gas (2.5 atm) we demonstrate tunability of the composition and morphology of the Ti TLIPSS protrusions. In particular, Raman spectroscopy revealed formation of TiN together with dominating TiO_2_ (rutile phase) in the TLIPSS protrusions produced in the nitrogen-rich atmosphere.

## 1. Introduction

Laser-induced periodic surface structures (LIPSS) have been intensively studied for several decades; however, there is still a room for investigating new aspects of this phenomenon. LIPSS are a periodic relief formed on the surface of solids due to the nonlinear absorption of high-energy laser pulses with multiple lines forming simultaneously within a focal spot [1,2]. An indefinitely large area can be covered with LIPSS by laterally scanning the surface with a laser beam. Various theoretical approaches have been applied to explain the LIPSS occurrence, including the scattering-interference mechanism, excitation of surface electromagnetic waves (polaritons, plasmons), counter-propagating surface plasmons, and material reorganization [3,4,5]. Although a comprehensive theoretical picture is still in development, LIPSS attract research attention due to the remarkable simplicity and reliability of the single-step technique of patterning the surface with subwavelength periodic features suggesting a wide range of possible applications, such as surface colorization, reflection/transmission tuning, wettability and friction control, security marking, energy storage and solar cells efficiency enhancement, biosensing, living cell growth and adhesion control, and more [6,7,8,9,10,11,12,13,14,15].

Depending on the material used, as well as laser irradiation parameters, LIPSS of various types can be fabricated. They can be classified by a period Λ relative to the wavelength of laser light λ, namely low-spatial-frequency LIPSS (LSFL) with Λ ≲ λ and high-spatial frequency LIPSS (HSFL) with Λ < λ/2 [1,16,17]. Both types can have orientation either parallel or perpendicular to the light polarization. Apart from their spatial characteristics, LIPSS can be classified by the formation process, which can be ablative or thermochemical. The former is due to material removal, which leaves periodic grooves on the irradiated surface. Many papers in the field of laser-induced periodic structures are focused on LIPSS formed through ablation, which have been studied since the first observation of LIPSS by Birnbaum in 1965 [18]. The occurrence of “ablative” LIPSS has been reported for metals, semiconductors and dielectrics [18,19,20,21,22,23,24]. In turn, the thermochemical mechanism is a chemical reaction of the irradiated material with the ambient environment, resulting in the formation of, for example, periodic oxide protrusions parallel to the laser polarization. Other mechanisms observed in the experiments include material reorganization, hydrodynamic instabilities, the reduction of graphene oxide, and phase transition [5,25,26,27,28].

Thermochemical LIPSS (TLIPSS) has gained a lot of research interest relatively recently, first obtained by Camacho-López et al. upon irradiation of Ti films with nanosecond laser pulses [29]. Later, Öktem et al. demonstrated the formation of extremely uniform TLIPSS on Ti films over large areas owing to the presence of positive and negative feedback [3]. TLIPSS fabrication by femtosecond (fs) laser pulses on a wide range of materials have been studied, including Ti, Cr, Hf, and Si reporting a rich variety of morphologies and chemical composition [3,29,30,31,32]. TLIPSS formation is usually explained by the interference between the waves scattered from surface defects and the incident part of the pulse, and the resulting interference maxima are oriented along the polarization. Intensity patterns create the corresponding periodic temperature distribution on the surface, which governs the thermochemical reaction, e.g., oxidation. With each pulse, growing oxide protrusions act as scatterers providing positive feedback for the process, increasing the uniformity of orientation of the structure. A thermochemical reaction continues if there are enough free oxygen molecules available at the material interface, but when the size of the oxide inclusions is so large that very little oxygen can migrate through it, the process halts. This constitutes negative feedback providing high uniformity of the width and height of the lines. The regime of TLIPSS fabrication has an energy threshold: the laser fluence should be less than a certain level to avoid ablation but high enough to start the thermochemical reaction [33].

As it is a multi-pulse mechanism of the formation, the speed of the sample surface scanning and pulse repetition rate influence the properties of the resulting TLIPSS. Direct dependency of the orientation on the light polarization demonstrates flexibility of this surface structuring method allowing dynamic change of the TLIPSS direction during the fabrication process by rotating the linear polarization [34]. Additionally, the period of the structure depends on the irradiation wavelength being somewhat less than λ (LSFL). Thus, by selecting the laser wavelength, one can obtain TLIPSS with the desired periodicity, which is attractive for practical applications. Most of the previous research works on TLIPSS fabrication have been performed using laser sources with λ in the near-IR spectral range, e.g., 800, 1030, and 1050 nm [3,30,35,36]. The TLIPSS formation on 320-nm thick Ti films with nanosecond laser pulses at λ = 532 nm has been reported previously; however, the structure uniformity was rather low [29].

In this paper, the formation of the TLIPSS on the Ti film surface was systematically studied upon fs-laser processing in air, a vacuum and a nitrogen-rich atmosphere using near-IR (1026 nm), visible (513 nm) and UV (256 nm) laser wavelengths. Ti was chosen for TLIPSS recording as a practically relevant material exhibiting high thermochemical activity allowing efficient oxidation.

## 2. Materials and Methods

TLIPSS were produced using fs-laser (pulse duration of 230 fs) pulses with the wavelengths λ of 1026, 513, and 256 nm at pulse repetition rate *f* of 50–200 kHz. The sample scanning speed *v* was varied within the range of 1–500 µm/s to cover different regimes of the TLIPSS formation. Laser fluence *F* was another parameter that was varied in the experiments. The samples under study were a two-layer Ti/α-Si film deposited by magnetron sputtering on a glass substrate with the Ti film being the top layer with a thickness of 90 or 180 nm. The α-Si bottom layer with a fixed thickness of 250 nm was used to avoid possible migration of oxygen from the substrate.

**Figure 1:**
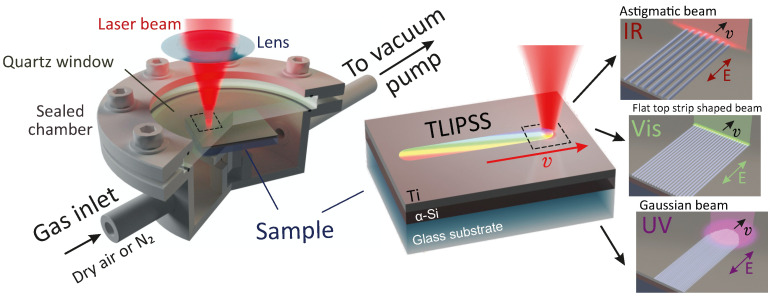
Schematically illustrated experimental setup for the TLIPSS formation using IR, visible and UV fs-laser radiation.

When using the IR laser pulses (λ = 1026 nm), the radiation was focused by a cylindrical lens yielding in an elliptical focal spot with an axis ratio of ≈1/10 and a width along the long side of 150 µm (Figure 1). To achieve the highest possible TLIPSS uniformity, at λ = 513 nm, we used a projection lithography to generate a 1-µm width 30-µm long flat-top stripe-shaped laser beam (see details in [37]). Such laser beams allow an increase in the surface patterning rate and improve the TLIPSS regularity owing to the reduced size of the stitching area, while requiring relatively higher pulse energies to achieve the fluence necessary for TLIPSS formation. Finally, the fourth-harmonic radiation (λ = 256 nm) was focused with a UV coated fused silica Bi-convex lens (focal distance of 20 mm) to yield in a 2-µm 1/e-diameter laser spot with an ordinary Gaussian-shaped lateral intensity profile. The samples were placed in a vacuum chamber, which had connected tubes for evacuating/pumping gas and a glass window for laser radiation (see Figure 1). For IR radiation λ = 1026 nm, laser processing was carried out under air, vacuum (a pressure down to 10^−2^ atm) and nitrogen-rich atmospheres (a pressure of 2.5 atm).

Surface morphology of the obtained structures was preliminary analyzed by a Hitachi S3400N scanning electron microscope (SEM), while more detailed analysis of selected surface areas was performed using a high-resolution Carl Zeiss Ultra 55+ SEM. To study the inner structure of the formed TLIPSS, cross-section cuts were prepared by focused ion beam (FIB) milling at Carl Zeiss CrossBeam 1540 apparatus following their visualization by SEM. The TLIPSS were coated with a 200-nm thick protective Ag film prior to FIB milling. Two-dimensional discrete Fourier transform (DFT) was applied to SEM images to calculate the TLIPSS period and analyze their uniformity. Chemical composition and crystallinity of the structures were studied by Raman spectroscopy. Raman spectra were measured using a laboratory-built experimental setup based on an SP-2500i monochromator and Spec 10:256E/LN CCD detector. Raman scattering was pumped by a laser radiation with a central wavelength of 532.1 nm and power less than 1 mW to avoid photo-thermo-modification of the TLIPSS properties. Measurements were performed in the backscattering regime using an objective with a numerical aperture of 0.75, which gave a focal spot of ∼1 µm diameter. A neon discharge lamp was used for calibration.

Finite-element frequency-domain calculations were carried out using COMSOL Multiphysics software. We considered a monochromatic linearly polarized wave with λ = 256, 513 or 1026 nm, which irradiates the air–Ti surface containing the TiO_2_ protrusion buried inside Ti film normally. The polarization direction of the incident wave was oriented along the protrusion. Three-dimensional geometry of the protrusion was modeled using experimental data information obtained from the SEM image of the FIB cuts.

## 3. Results and Discussion

**Figure 2:**
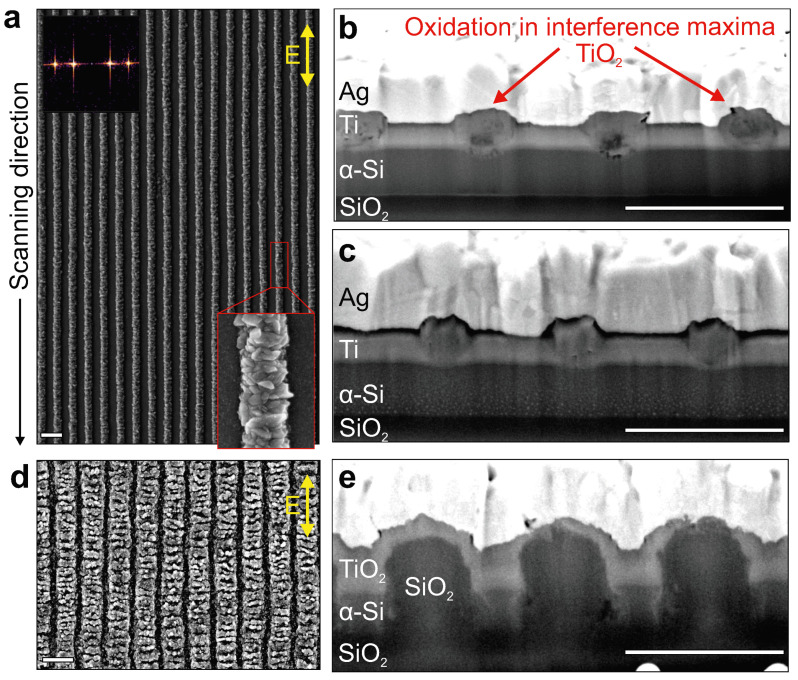
(**a**) Top-view SEM image of TLIPSS structures produced above the 90-nm thick Ti film in air at λ = 1026 nm, *F* = 90 mJ/cm^2^ and *v* = 1 µm/s. A double arrow indicates the polarization direction. Top inset provides 2D-DFT image of the corresponding SEM image. Bottom inset shows the enlarged view of the single TiO_2_ protrusion revealing its nanocrystalline structure. (**b**,**c**) SEM images of cross-sectional FIB cuts made perpendicularly to the TLIPSS orientation. Both types of TLIPSS were produced at fixed *v* = 1 µm/s and laser fluences *F* = 90 (**b**) and 45 (**c**) mJ/cm^2^. (**d**) Top-view SEM image of TLIPSS structures produced at *F* = 110 mJ/cm^2^ and *v* = 1 µm/s. (**e**) SEM image of the cross-sectional FIB cuts of the TLIPSS presented in (**d**). Scale bar in the images indicates 1 µm.

Figure 2 demonstrates the formation of highly regular TLIPSS on the surface of 90-nm thick Ti film upon its processing in air with IR laser radiation (λ = 1026 nm, *F* = 90 mJ/cm^2^, *v* = 1 µm/s). Two-dimensional DFT analysis of the SEM images indicates the characteristic TLIPSS period of 853 ± 20 nm. The TLIPSS are oriented along the polarization of the laser radiation, as is expected from the underlying ordering mechanism coming from the constructive interference of the incident laser pulses with the radiation scattered by the surface irregularities. Each formed surface protrusion with a width of ≈400 nm consists of TiO_2_ nanocrystallites with a random orientation as can be seen from the magnified SEM image (Figure 2a). Raman spectroscopy revealed that the protrusions are mainly composed of rutile phase with its characteristic Raman bands appearing at 445 and 610 cm^−1^ (see discussion below). More insight into the morphology of the produced TLIPSS was obtained from SEM visualization of the transverse cross-sectional FIB cuts (see Methods section). In particular, the chosen laser-processing parameters allow the TiO_2_ to grow from the top of the Ti film toward the Ti/Si interface. The rutile starts to grow from the top of the Ti film owing to thermal-induced interaction with oxygen. Owing to porous packing of rutile nanocrystals as well as rather large Pilling and Bedworth ratio $R_{PB}=1.78$ for titanium and its oxide (rutile) [38], the produced protrusions occupy a larger volume compared to that of the pristine film. Once the rutile reaches the Ti/Si interface, deformation of the α-Si film surface is observed below the protrusions. It is worth noting that the laser-processing regime allows the regulation of the geometry of the TiO_2_ fraction as illustrated by the SEM image of the additional FIB cut made through the TLIPSS produced at same scanning speed but at twice as low fluence (Figure 2c). As can be seen, the protrusions obtained at lower fluence have smaller thickness, leaving the α-Si sub-layer almost intact, while their lateral width also reduced to ≈320 nm.

Larger fluence *F* = 110 mJ/cm^2^ allows the initiation of the oxidation process even in the underlying α-Si film (see Figure 2d,e). Oxidation occurs just below the surface protrusions and affect the entire thickness of the α-Si film resulting in the formation of rather large SiO_2_ pillars with a height of ≈500 nm. SEM contrast does not allow the distinguishing between the pillars and underlying glass substrate, particularly confirming the deduction regarding composition of the obtained morphology. Moreover, the surface of the Ti film appears to be completely covered by the oxide TiO_2_ layer as the Ti material can hardly be identified on the corresponding SEM image of the cross-sectional cut (Figure 2e). Closer investigation of the surface morphology of the TLIPSS produced under such processing conditions reveals the periodic height modulation of the protrusions oriented perpendicularly to the polarization direction. The averaged modulation period was found to be 270 ± 62 nm. This behavior can be explained by either electromagnetic [39] or hydrodynamic scenarios [32,40] whose clarification is out of the scope of this paper. In general, uniform and regular TLIPSS appeared within the broad range of applied fluences *F* (45–90 mJ/cm^2^) when a rather low scanning speed (1–25 µm/s) is preserved. For higher scanning rates the regularity of the TLIPSS gradually deteriorates. At the highest laser fluence of *F* = 130 mJ/cm^2^, the central area of the writing track becomes overexposed leading to a doubling of the spatial frequency of TLIPSS, appearing as additional oxide protrusions that grow in between the main TiO_2_ ridges. This agrees with the previously reported results [33].

**Figure 3:**
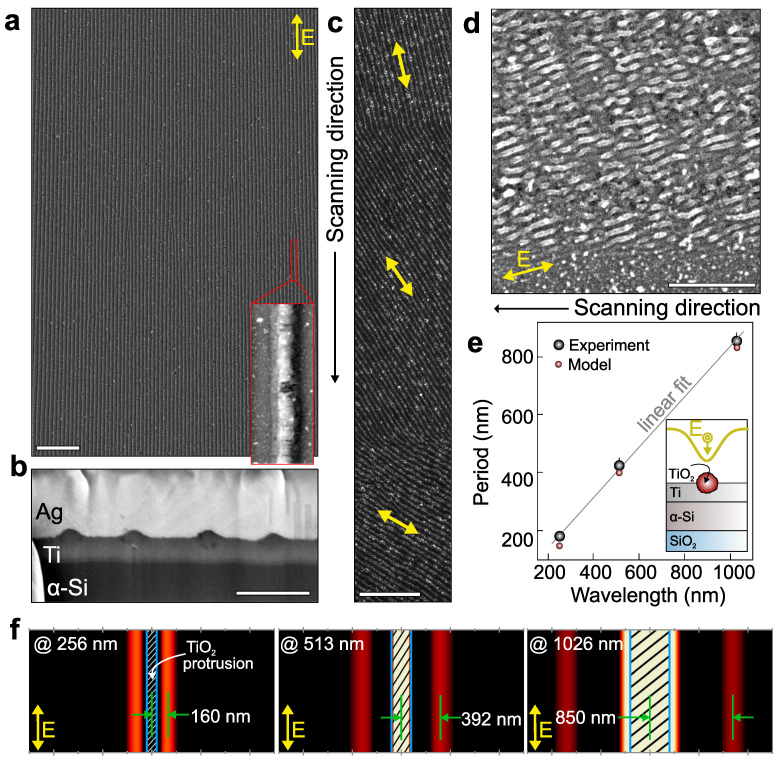
(**a**) Top-view SEM image of TLIPSS structures produced above the 90-nm thick Ti film in air at λ = 513 nm (*F* = 50 mJ/cm^2^, *v* = 3 µm/s). Inset shows the enlarged view of the single TiO_2_ protrusion. (**b**) SEM images of cross-sectional FIB cut made perpendicularly to the TLIPSS orientation. (**c**) Top-view SEM image of similar TLIPSS produced by step-like variation of the polarization direction in the process of sample scanning with a laser beam. (**d**) SEM image of the TLIPSS produced at λ = 256 nm (*F* = 35 mJ/cm^2^, *v* = 1 µm/s). A double-headed arrow in the SEM images indicates the polarization direction. Scale bars in the SEM images indicate 5 µm (**a**,**c**) and 500 nm (**b**,**d**). (**e**) Measured (black markers) and calculated (red markers) averaged TLIPSS period vs. incident laser wavelength used for their fabrication. Inset: schematic illustration of the modeled geometry. (**f**) 2D intensity profiles calculated near the Ti surface containing the central TiO_2_ protrusion at different incident laser wavelengths. Color scale was adjusted for better visibility. Shaded rectangles indicate the area occupied by the protrusion.

Since the TLIPSS ordering is caused by the interference of the incident radiation with that scattered by the surface roughness, the period of the surface morphology should scale with λ opening pathways for achieving subwavelength gratings with a visible/UV radiation. Meanwhile, no experimental studies have been carried out so far to unveil the formation of the regular TLIPSS on the Ti surface within this practically relevant spectral range. We started by fabricating TLIPSS with second-harmonic (λ = 513 nm) laser pulses (see Methods for details). The SEM image in Figure 3a demonstrates the formation of extremely uniform TLIPSS with their period of 416 ± 17 nm at *v* = 3 µm/s and *F* = 50 mJ/cm^2^. The TLIPSS protrusions are oriented along the polarization direction indicating similar interference-based feedback underlying the grating ordering. For the chosen laser-processing parameters, the TiO_2_ protrusions exhibit smoother surface morphology (being compared to the TLIPSS produced with IR laser pulses) and affect ≈40 nm of the Ti film thickness, as can be revealed from the SEM image of the cross-sectional FIB cut (Figure 3b). The averaged width of the protrusions was found to be ≈170 nm (see inset in Figure 3a) indicating that thermal-induced oxidation process at the air–Ti interface can be efficiently confined using tightly focused radiation (which is achieved by shorter laser wavelengths in this case). It is worth noting that higher pulse energies result in cracking of the TLIPSS protrusions accompanied by the redeposition of the removed TiO_2_ material from the formed cracks (not shown here). As with the TLIPSS recording with IR laser radiation, faster scanning speed worsens the grating regularity owing to deterioration of the positive interference-based feedback. Regular TLIPSS have been found to form at *F* = 50–70 mJ/cm^2^ and *v* of up to 5 µm/s.

Figure 3c illustrates how the TLIPSS orientation follows the step-like change of the polarization direction upon continuous scanning of the sample with a laser beam (from top to bottom direction). As can be seen, the change of the polarization direction results in fast reorientation of the TLIPSS protrusion without substantial degradation of the ordering. This highlights the TLIPSS as a promising and scalable technology for the fabrication of flat optics, including polarizers and birefringent optical elements. Although regular TLIPSS were formed on the Ti film surface at a relatively low scanning speed *v*, the patterning rate can be increased using MHz pulse repetition rates to preserve constant pulse overlap at elevating scanning speed. It is worth noting that similar highly regular TLIPSS were previously formed on the surface of other oxidizing metals (as Cr and Hf [31,41]) at much faster scanning rates (up to 2000 µm/s).

To further reduce the TLIPSS period, sample processing was carried out using fourth-harmonic (λ = 256 nm) laser pulses. These experiments were performed with an ordinary Gaussian-shaped laser beam owing to complexity of the precise beam shaping in this spectral range and low output intensity at this wavelength. The laser processing was undertaken by scanning the sample along a snake-like trajectory keeping the 0.5-µm span between the parallel scans. The morphology of the Ti film processed at maximal available fluence *F* = 35 mJ/cm^2^ and scanning speed of *v* = 1 µm/s is shown in Figure 3d revealing formation of the TLIPSS with the characteristic period of 182 ± 22 nm.

Again, the protrusions are arranged along the polarization direction and exhibit scalable reduction of the width down to ≈100 nm. Meanwhile, the regularity of the UV-printed TLIPSS is much worse compared to those for surface morphologies produced with IR/visible laser radiation. This particularly originates from the small size of the laser spot causing grating defects in the multiple stitching areas. Moreover, Ti and TiO_2_ exhibit much stronger absorption at λ = 256 nm compared to that for IR and the visible spectral range. This allows the incident UV pulses to cause solid–liquid transition in the irradiated material more efficiently (compared to visible and IR radiation) and can lead to potential competition between different mechanisms of the morphology evolution: (i) ordered oxidation and formation of the protrusions in the interference maxima vs. (ii) random material ablation via ejection of the molten droplets and their redeposition onto the film surface. This laser-generated surface debris covering the non-processed surface area is clearly seen in the SEM image (Figure 3d) and was almost absent for optimized processing regimes at λ = 1026 and 513 nm. Such an ablative regime is also expected to deteriorate TLIPSS ordering. Figure 3e shows that the averaged TLIPSS period linearly scales with the wavelength of the incident laser radiation used for their fabrication, confirming the pure electromagnetic origin of the surface morphology ordering.

Finite-element frequency-domain calculations were undertaken to explain scalability of the TLIPSS period with the laser wavelength. For simplicity, we considered the isolated TiO_2_ protrusion irradiated from the top by the electromagnetic wave with the wavelengths λ matching those used in the experiments (see Methods for details). Indeed, the geometry of the modeled protrusion as well as its composition and structure will affect the calculated results for a given wavelength. Although the general 3D morphology of the protrusion (height and width) can be reproduced precisely from the SEM images of the cross-sectional FIB cuts, the inner structure defined by random arrangement of the nanocrystallites is more complicated to address in the model [30]. To take into account the porous structure of the TiO_2_ protrusion, we modeled its complex refractive index as $n=\left(1-m\right)n_{{\mathrm{TiO}}_{2}}+mn_{air}$, where *m* is the porosity factor, $n_{{\mathrm{TiO}}_{2}}$ and $n_{air}$ are the refractive indices of solid TiO_2_ and air, respectively.

Figure 3f presents the normalized intensity maps calculated at the Ti–air interface in the vicinity of the TiO_2_ protrusion. As can be seen, for all modeled wavelengths, the intensity maxima appear on both sides of the protrusion at a certain distance from the ridge center. The intensity maxima resulting from the interference of the incident and the scattered waves are expected to serve as the sources for the formation of the subsequent protrusion via thermal-induced oxidation. Thus, the distance between the protrusion center and the interference maxima can be used to roughly assess the expected TLIPSS period for each simulated wavelength λ. It is worth noting that for *m* = 0 the model predicts the TLIPSS period of 160 and 392 nm at λ = 256 and 513 nm, respectively, providing reasonable matching with the experimentally observed TLIPSS periods (≈180 and 420 nm, respectively) and indicating a negligible porosity of the formed protrusions (see Figure 3e). Better matching between calculated and experimental results can be achieved by slightly adjusting the modeled porosity factor. However, similar calculations performed at *m* = 0 and λ = 1026 nm predict the substantially smaller TLIPSS period of ≈700 nm compared to those observed experimentally (≈850 nm). This can indicate a strong mismatch between the tabulated values of the TiO_2_ refractive index and the real refractive index of the porous TiO_2_ protrusions composed of randomly arranged nanocrystallites (as experimentally observed at λ = 1026 nm; see Figure 2a). Systematic simulations show that good matching between the calculated and measured TLIPSS periods can be achieved at *m* = 0.5, particularly confirming the abovementioned issue.

**Figure 4:**
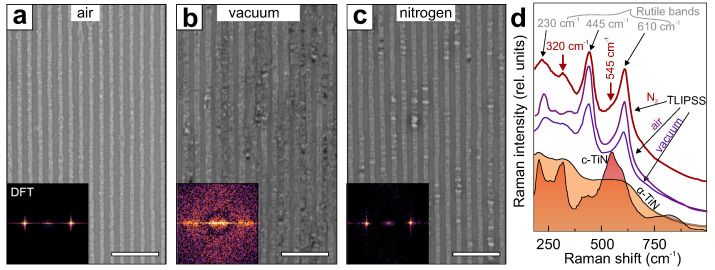
(**a**–**c**) SEM images of the TLIPSS formed on the surface of 180-nm thick Ti film in air (**a**), vacuum (**b**) and nitrogen-rich atmosphere (**c**). Fixed laser-processing conditions were used for TLIPSS fabrication: *f* = 200 kHz, *F* = 70 mJ/cm^2^, *v* = 1 µm/s. Scale bar indicates 4 µm. Insets show DFT images taken from the corresponding SEM images. (**d**) Averaged Raman spectrum of the TLIPSS produced in the nitrogen-rich atmosphere (red curve), air (purple curve) and vacuum (blue curve). Raman spectra of amorphous and crystalline TiN are provided for comparison. Characteristic bands associated with TiO_2_ (rutile) and TiN are also indicated.

As the growth of the parallel TLIPSS protrusions originates from a thermally stimulated chemical process of the Ti film oxidation, their morphology and composition should be regulated by the ambient atmosphere, i.e., the content of oxygen molecules or other gases in the surroundings. To study this effect, TLIPSS fabrication with IR laser pulses was undertaken in a vacuum and in a nitrogen-rich atmosphere (see Methods for details). Figure 4 compares the morphology of the TLIPSS formed at the following processing parameters: *f* = 200 kHz, *F* = 70 mJ/cm^2^, *v* = 1 µm/s, in air, a vacuum and a nitrogen-rich atmosphere (see Methods). As can be seen, for the vacuum environment TLIPSS regularity worsens, while some additional oxide material is formed between the protrusions (Figure 4b). This is also illustrated on a 2D-DFT map indicating a noise within a wide range of spatial frequencies (inset in Figure 4b). The characteristic period of the vacuum-formed TLIPSS slightly increases to 943 ± 30 nm (in comparison to those for TLIPSS formed in air ≈870 ± 21 nm). A similar period (≈940 ± 23 nm) and slightly better ordering was found for TLIPSS formed in the nitrogen-rich atmosphere (Figure 4c). The difference of the TLIPSS period produced in different ambient environments can be explained by the varying geometry of the TiO_2_ protrusions, their inner structure (porosity factor), and chemical composition affecting the refractive index.

Since TLIPSS are formed as a result of a thermally stimulated chemical reaction, the presence of high concentrations of nitrogen can lead to the formation of nitrides along with oxides. Raman spectroscopy was used to probe the chemical composition of the TLIPSS formed in the nitrogen-rich atmosphere, as well as in air and a vacuum. Typical Raman spectra of the TLIPSS averaged over multiple surface sites revealed several characteristic bands appearing at 230, 320, 445 and 610 cm^−1^ (Figure 4d). Apart from the band at 320 cm^−1^, the remaining ones can be attributed to characteristic vibration modes of rutile [42,43] indicating that the oxidation process dominates the protrusion formation, even in the case of TLIPSS fabrication in the nitrogen-rich atmosphere that could contain a certain amount of the oxygen molecules. Similar bands can also be identified in the typical Raman spectra of the TLIPSS formed in the air and vacuum atmospheres (purple and blue curves in Figure 4d). The band at 320 cm^−1^ is characteristic of TiN [44] as can be also seen from the analysis of the reference Raman spectra measured from the crystalline TiN substrate (c-TiN) as well as its amorphous film (α-TiN) obtained by magnetron deposition onto a glass substrate (Figure 4d). This band is noticeable in the Raman spectrum of TLIPSS formed in the nitrogen-rich atmosphere and is undiscerned in the spectra measured for morphologies produced in air and vacuum environments. A small amount of the formed TiN can be also identified in the spectrum through the low-intensity band at 545 cm^−1^ particularly screened by the main rutile band at 610 cm^−1^.

## 4. Conclusions

In conclusion, the formation of thermochemical laser-induced periodic surface structures on Ti films was demonstrated using near-IR (λ = 1026 nm), visible (λ = 513 nm) and UV (λ = 256 nm) fs-laser radiation. The surface morphologies with characteristic periods of ≈850, 420 and 180 nm were consequently produced using the abovementioned fs-laser wavelengths demonstrating linear dependence of the TLIPSS periodicity versus the radiation wavelength. Full-wave electromagnetic simulations provided valuable insight into the TLIPSS ordering mechanism, revealing that both the complex morphology and composition of the protrusions should be taken into account. Almost perfect regularity of the TLIPSS produced at near-IR and visible wavelength was found to deteriorate when UV radiation was applied for their recording. This can reflect competition between thermochemical oxidation and ablative liquid-phase material removal in the process of the TLIPSS formation upon UV exposure. Finally, we showed that TLIPSS regularity as well as morphology and composition of the protrusions can be regulated not only by laser radiation wavelength but also by the ambient environment. For example, processing in a vacuum was found to cause deterioration of the TLIPSS regularity owing to degradation of the oxidation efficiency. Moreover, Raman spectroscopy revealed the protrusions with a complex TiO_2_/TiN composition in the TLIPSS produced in the nitrogen-rich atmosphere. The performed experiments showed that for both a vacuum (10^−2^ atm) and a nitrogen-rich atmosphere, the oxidation process still governs the TLIPSS protrusion formation, inviting the exploration of high-vacuum processing conditions, which will become a subject of our forthcoming studies.

## Data Availability

The data presented in this study are available on request from the corresponding author.
